# Female northern grass lizards judge mates by body shape to reinforce local adaptation

**DOI:** 10.1186/s12983-020-00367-9

**Published:** 2020-08-04

**Authors:** Kun Guo, Chen Chen, Xiao-Fang Liang, Yan-Fu Qu, Xiang Ji

**Affiliations:** Jiangsu Key Laboratory for Biodiversity and Biotechnology, College of Life Sciences, Nanjing Normal University, Nanjing, 210023 Jiangsu China

**Keywords:** Geographical distance, Genetic differentiation, Local adaptation, Mate preference, Morphology, Structural equation model

## Abstract

**Background:**

Identifying the factors that contribute to divergence among populations in mate preferences is important for understanding of the manner in which premating reproductive isolation might arise and how this isolation may in turn contribute to the evolutionary process of population divergence. Here, we offered female northern grass lizards (*Takydromus septentrionalis*) a choice of males between their own population and another four populations to test whether the preferences that females display in the mating trials correlate with phenotypic adaptation to local environments, or to the neutral genetic distance measured by divergence of mitochondrial DNA sequence loci.

**Results:**

Females showed a strong preference for native over foreign males. Females that mated with native versus foreign males did not differ from each other in mating latency, or copulation duration. From results of the structural equation modelling we knew that: 1) geographical distance directly contributed to genetic differentiation and environmental dissimilarity; 2) genetic differentiation and environmental dissimilarity indirectly contributed to female mate preference, largely through their effects on morphological divergence; and 3) females judged mates by body shape (appearance) and discriminated more strongly against morphologically less familiar allopatric males.

**Conclusions:**

Local adaptation rather than neutral genetic distance influences female mate preference in *T. septentrionalis*. The tendency to avoid mating with foreign males may indicate that, in *T. septentrionalis*, local adaptations are more valuable than genetic novelties. Our results highlight the importance of comprehensive studies integrating ecological, molecular and behavioral approaches to understand population divergence in female mate preferences as the consequence of local adaptations.

## Background

Geographically separated populations accumulate genetic and phenotypic differences through genetic drift and/or adaptation to local environmental conditions [1]. The roles of local adaptation and consequent divergent selection between environments on the evolution of mate preferences are widely recognized in the literature and have been documented in diverse taxonomic groups such as snails [2], crustaceans [3, 4], insects [5–8], fish [9–14], amphibians [1, 15–17], reptiles [18], birds [19, 20] and mammals [21, 22]. From previous studies on mate preference in females, which account for the vast majority of published papers focusing on this issue, we know the following. First, mate preferences are often evaluated with respect to behaviors associated with their intentions of rejecting and/or accepting males rather than actual copulations with males [18]. Second, local adaptations are more valuable than genetic novelties in most of the species studied thus far, as revealed by the fact that females of these species prefer to mate with native (local) over foreign males [2]. To our knowledge, the Trinidadian guppy *Poecilla reticulata* [9], the sailfin molly *Poecilia latipinna* [13], the striped mouse *Rhabdomys pumilio* [22], the Pacific blue-eye *Pseudomugil signifer* [14] and the rainwater killifish *Lucania parva* [11] represent the exceptions of species where female mate preference for native males is strong in some populations but not in others, or asymmetric in different populations. Third, comprehensive studies integrating molecular, ecological, phenotypic and behavioral approaches to show changes in mate preference correlated with local adaptation are rare. To our knowledge, such studies have been performed only in *P. signifer* [14], the Allegheny mountain salamander *Desmognathus ochrophaeus* [17] and the fire salamander *Salamandra salamandra* [15] where female mate preferences depend either on local adaptation rather than neutral genetic distance (*S. salamandra*), or on how genetically and geographically separated populations are from one another (*P. signifer* and *D. ochrophaeus*).

Divergence among populations in mate preferences is a crucial step of population differentiation and potential speciation because it may lead to premating reproductive isolation [23, 24]. Identifying the factors that contribute to such a divergence is therefore important for understanding of the manner in which premating reproductive isolation might arise and how this isolation may in turn contribute to the evolutionary process of population divergence and incipient speciation [25]. However, as the roles of individual factors in mate assessment by males or females are not mutually exclusive, disentangling their effects on mate preferences is difficult. Fortunately, the recent progress in statistical methods for spatial data analysis and the increasing availability of high-resolution geographical and environmental data layers make it possible to evaluate the relative contributions of the factors to population divergence in mate preferences.

**Fig. 1:**
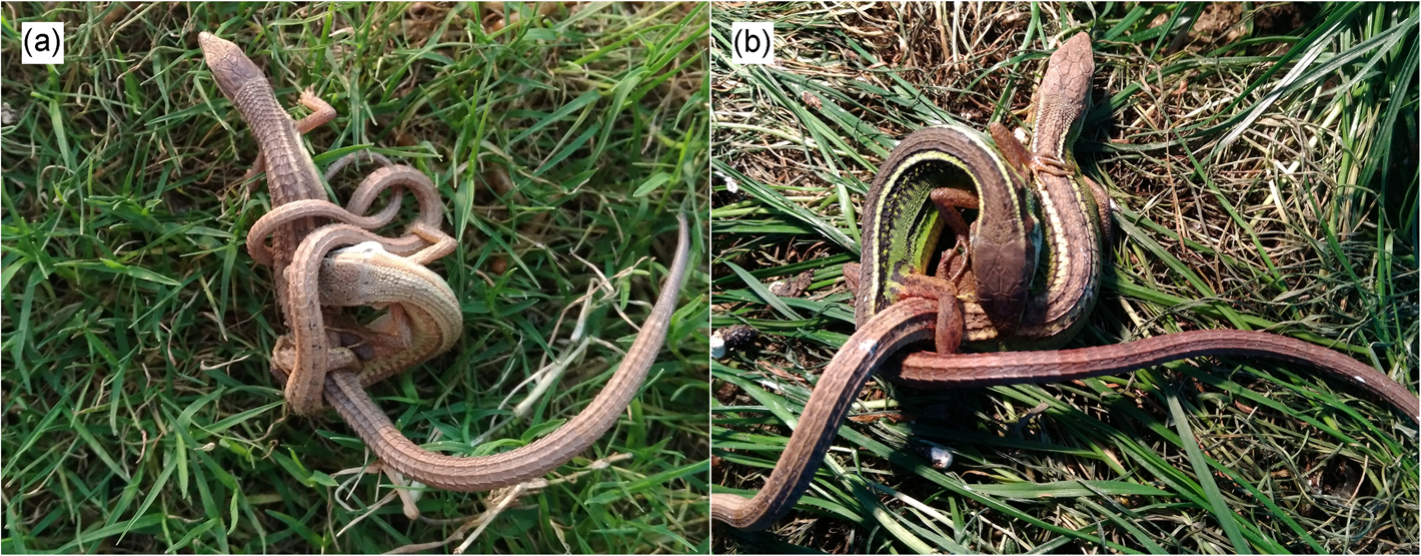
The body posture of heterospecific **a**, between male *T. septentrionalis* and female *T. sexlineatus*) and conspecific homosexual **b**, between males of *T. septentrionalis*) mating partners

The northern grass lizard (*Takydromus septentrionalis*) is a multiple-clutched oviparous lacertid lizard endemic to China and has a distributional range covering the central and southeastern parts of the country [26]. The lizard consists of three divergent lineages, with isolation by distance known to be the main cause of genetic divergence [27]. Proximate factors are less important determinants of spatio-temporal variation in life-history traits (e.g., size at maturation, adult size, clutch size, clutch frequency and egg size) than are genetic influences [28–31]. Adults can have up to three distinct color morphs within a population: morph 1 is shared by both sexes, with a yellowish-white longitudinal stripe between brown dorsal and green lateral surfaces; morph 2 is a typical male coloration, with morph 1 decorated with numerous black speckles on the lateral surface; morph 3 is a typical female coloration, with brown dorsal and green lateral surfaces connected directly (Additional file 1). Although small adults do mate less frequently than large ones, the lizard does not show size-assortative mating [32]. Males are the less choosy sex, as revealed by three lines of evidence. First, they can be easily induced to mate at extreme phylogenetic distances, either with conspecific females from other populations, or even with heterospecific (but congeneric) females (e.g., *T. sexlineatus*) (Fig. 1a), which is an extreme example of outcrossing. Second, males are often found to mate with one another in a manner adopted by heterosexual mating partners (Fig. 1b). Third, female receptivity rather than male sexual coercion (and thus forced insemination) has a direct role in determining mating success, and females are more receptive to mate within 2 d post-laying and much less so thereafter [32]. Here, we describe a study offering each post-laying female *T. septentrionalis* 10 males, two from each of five geographically separated populations (see below for details), to test whether the species also shows a female mate preference for local males and, if so, whether the preferences that females display in the mating trials correlate with phenotypic adaptation to local environments, or to the neutral genetic distance measured by divergence of mitochondrial DNA sequence loci.

## Materials and methods

### Animal collection and maintenance

**Fig. 2:**
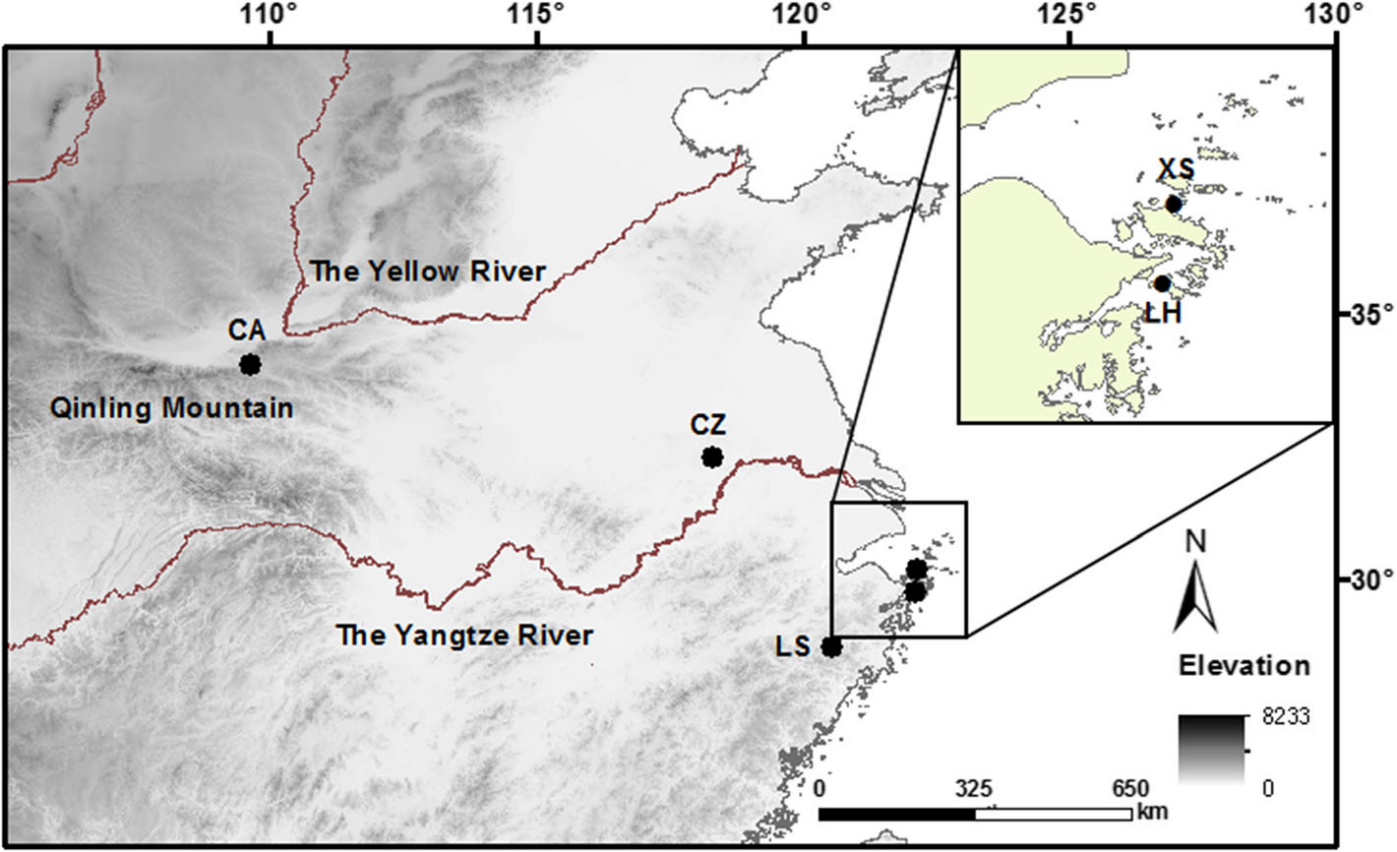
Locations of the five *Takydromus septentrionalis* populations from which we collected adult lizards. Three mainland populations are in Chang’an (CA), Chuzhou (CZ) and Lishui (LS); two island populations are in Liuheng (LH) and Xiushan (XS), Zhoushan Islands

We collected adult *T. septentrionalis* in early April 2015 from five populations that are between 50 and 1340 km apart: three mainland populations in Chang’an (CA: 34°01′N, 108°58′E), Chuzhou (CZ: 32°11′N, 118°11′E) and Lishui (LS: 28°26′N, 119°55′E); two island populations in Liuheng (LH: 29°43′N, 122°08′E) and Xiushan (XS: 30°10′N, 122°10′E), Zhoushan Islands (Fig. 2). Healthy lizards without visible ectoparasites were brought to our laboratory within a week of capture. A total of 150 males of morph 1 shared by both sexes, 30 from each of five populations, and 100 LS females yet to lay their first clutch were used in this study. Each male was painted with a unique Arabic number on the belly for identification. Measurements taken for each lizard included snout-vent length (SVL), abdomen (or axillo-groin) length (AL, between the inserting points of the fore- and hind-limbs), head length (HL, from the snout to the anterior edge of the external auditory meatus) and head width (HW, taken at the posterior end of the mandible). Males from the five populations differed morphologically (both body size and body shape): the mean SVL was greatest in the CA population and smallest in the LS, LH and XS populations; the SVL-adjusted mean AL was greater in the CZ and CA populations than in the LS, LH and XS populations; the SVL-adjusted HL was greatest in the LS population and smallest in the CA population; the SVL-adjusted mean HW was greater in the LS and LH populations than in the XS, CZ and CA populations (Additional file 2).

Ten males (two randomly chosen from each of five populations) or 10 females from the LS population were housed in one 900 × 600 × 400 (length × width × height) mm cage with a soil substrate (~ 50 mm depth) covered with grass and pieces of clay tile. All cages were placed in a room inside which temperatures varied from 20 to 28 °C. Thermoregulatory opportunities were provided between 07:00–19:00 h by a 60 W full-spectrum lamp at one end of each cage to create a thermal gradient from room temperature to ~ 50 °C during the photophase. Mealworms (*Tenebrio molitor*), house crickets (*Achetus domesticus*) and water enriched with vitamins and minerals were provided daily. All lizards used in this study were released to the sites where they were originally captured in early August, soon after the breeding season.

### Mate choice trials

Only females that had just laid the first clutch were used to test their mate preference for native versus foreign males. We randomly moved a post-laying female into one all-male cage. Mating often took place in 90 min, and any female that did not mate with a male in 2 h was randomly moved into another all-male cage. We recorded mating latency (time from putting a female in an all-male cage to the beginning of copulation) and copulation duration (time from the beginning to the end of copulation) for each mated pair. Of the 100 females tested, 99 successfully mated in 2 d post-laying.

### Geographical, genetic, environmental and morphological differences

We calculated geographic distance for each pair of populations based on their latitude and longitude data using Geographic Distance Matrix Generator 1.2.3 [33]. We quantified genetic divergence (*F*st) for each pair of populations based on a fragment of mitochondrial DNA that was 1143 base pairs long [27]. We performed a principal components analysis (PCA) for the five populations to resolve two components (eigenvalues ≥1) from 22 environmental (three geographical and 19 climatic) variables (https://www.worldclim.org [34]), explaining ~ 92% of the variation in the original data (Additional file 3). We performed a PCA for 150 adult males from the five populations to resolve two components (eigenvalues ≥1) from three SVL-adjusted body-shape variables, explaining ~ 91% of the variation in the original data (Additional file 4). We calculated environmental and morphological divergence between each pair of populations as the difference in their mean PC1 scores.

### Statistical analyses

All statistical analyses were performed using Statistica 8.0 (StatSoft Inc., Tulsa, OK, USA). We used a *G* test to examine whether female mate preference was population-dependent. We used PCA scores to show environmental and morphological differences between populations. We used nonlinear estimation to show the frequency of matings in relation to geographic distance, genetic differentiation, environmental dissimilarity and morphological difference. We used one-way ANOVA or ANCOVA with SVL as the covariate to examine whether mating latency, mating duration and morphological traits measured differed among the five populations. We used mixed model ANOVA with population origin as the fixed factor and cage ID as the random factor to examine whether the distribution of male morphologies (PC1 scores) available to individual females in mate choice trials matched the overall distribution of male morphologies from the five populations. A Tukey’s *post-hoc* test was performed when necessary to find means that were significantly different from each other.

We performed statistical analyses for structural equation modelling (SEM) in AMOS 21.0 [35] to quantify the relative contributions of geographical distance, genetic differentiation, environmental dissimilarity and morphological difference to female mate preference, showing the paths and framework among the five variables. We constructed a priori models and tested the optimized one with a method unweighted least squares in SEM [36]. We used z-scores to standardize data, thereby controlling for the influence of dimensional differences among variables.

## Results

**Fig. 3:**
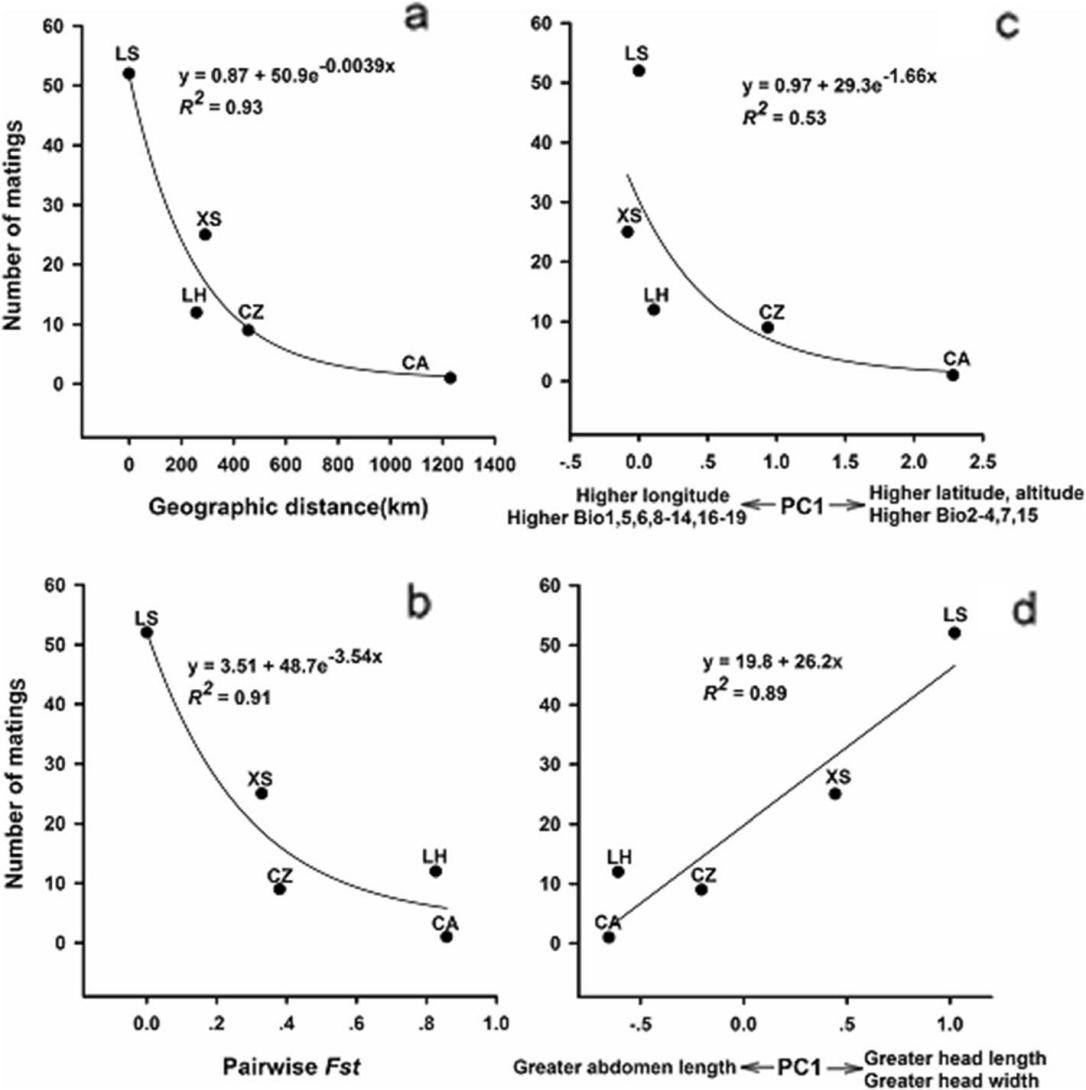
Frequency of matings in relation to geographical distance **a**, pairwise *F*st (a measure of genetic differentiation between populations, **b**, environmental dissimilarity **c** and morphological difference **d**. Regression equations and coefficients are given in the figure. See Fig. 2 for abbreviations for sampling locations

Population origin (*F*_4, 56_ = 37.52, *P* < 0.001) but not cage ID (*F*_14, 56_ = 1.39, *P* = 0.485) or the population × cage interaction (*F*_56, 75_ = 0.60, *P* = 0.975) was a significant source of morphological variation for males in 15 all-male cages. The lack of a significant cage effect suggested that the distributions of morphological traits in individual all-male cages did not deviate from the overall distribution of male morphologies from the five populations. Table 1 and Fig. 3 show the results of the female preference tests for males from native versus foreign populations. Females showed a strong preference for native over foreign males (*G* = 79.86, *df* = 4, *P* < 0.001). Of the 99 LS females mating in 2 d post-laying, 52 mated with males from their own population, 25 with XS males, 12 with LH males, 9 with CZ males, and 1 with a CA male. Females that mated with native versus foreign males did not differ from each other in mating latency (*F*_3, 94_ = 0.60, *P* = 0.620), or copulation duration (*F*_3, 94_ = 2.24, *P* = 0.089). The frequency of matings between females and males from different populations generally decreased at an ever-decreasing rate as geographical distance (Fig. 3a), genetic differentiation (Fig. 3b), or the male’s mean environmental PC1 score (Fig. 3c) increased, but at almost the same rate as the male’s mean morphological PC1 score increased (Fig. 3d).

**Table 1:** Descriptive statistics, expressed as mean ± SE and range, for female SVL, male SVL, mating latency and copulation duration. All females were from the Lishui population, and males from five geographically distinct populations

Male populations	*n*	Female SVL (mm)	Male SVL (mm)	Mating latency (min)	Copulation duration (min)
Lishui (LS)	52	65.4 ± 0.4 (60.0–73.7)	65.9 ± 0.3 (60.6–69.2)	23.6 ± 2.4 (1–90)	153.0 ± 7.0 (34–325)
Xiushan (XS)	25	66.4 ± 0.7 (60.0–71.9)	65.2 ± 0.3 (61.7–69.3)	19.0 ± 2.2 (6–55)	168.8 ± 9.5 (41–269)
Liuheng (LH)	12	64.6 ± 0.8 (60.4–68.5)	66.9 ± 0.7 (63.1–72.7)	15.0 ± 1.7 (9–25)	187.5 ± 16.5 (94–320)
Chuzhou (CZ)	9	64.3 ± 0.7 (61.0–68.3)	66.4 ± 1.6 (60.2–70.1)	17.0 ± 2.3 (6–26)	129.1 ± 13.3 (76–210)
Chang’an (CA)	1	61.1	79.8	30	140

**Fig. 4:**
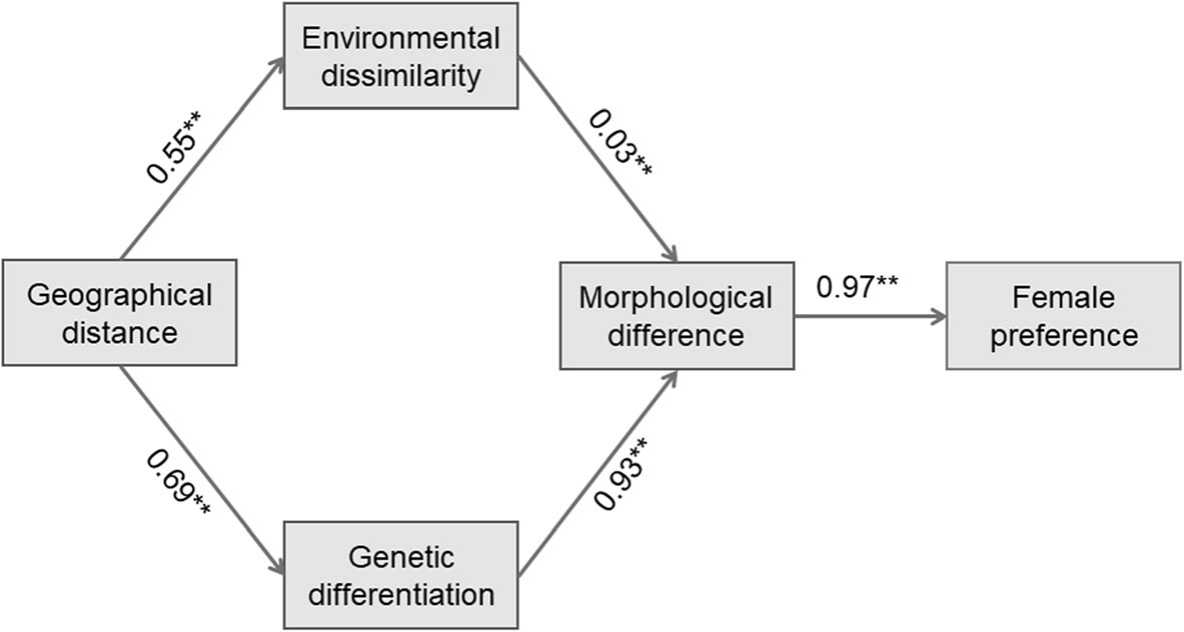
The most supported structural equation model for the roles of geographical distance, genetic differentiation, environmental dissimilarity and morphological difference in mate assessment by female *T. septentrionalis*. Numbers given in the figure represent the standard direct effects, and the symbol ** represents *p* < 0.01

All model-fit indices [CMIN = 0.010 (*df* = 15, *P* > 0.05), RMR = 0.003, GFI = 0.997, AGFI = 0.992, NFI = 0.995, RFI = 0.991] supported that the model and framework we selected were optimized (Fig. 4). From the results of SEM we knew the following. First, geographical distance directly contributed to genetic differentiation and environmental dissimilarity. Second, genetic differentiation directly contributed to morphological difference, and so did environmental dissimilarity although its role in shaping morphology was comparatively less substantive. Third, morphological difference directly contributed to female mate preference. Fourth, genetic differentiation and environmental dissimilarity indirectly contributed to female mate preference, largely through their effects on morphological divergence. Fifth, genetic and morphological divergence had greater total effects on female mate preference than did geographical distance and environmental dissimilarity (Additional file 5).

## Discussion

Males collected from the five allopatric populations differed morphologically and genetically. More specifically, they differed not only in body size but also in body shape (appearance) as revealed by the fact that SVL-specific mean values for AL, HL and HW differed among the five populations (Additional file 3), and their neutral genetic divergence generally increased as geographical distance increased [27]. In the scenario of maximizing genetic novelties, we would have expected female northern grass lizards to mate more frequently with foreign males, but this did not happen, even though such a preference could benefit females in leading to the production of genetically more different offspring. Instead, when offering LS females the choice between males from their own population and other four populations, we found that they were capable of distinguishing between native and foreign males and strongly discriminated against foreign males to facilitate population divergence or local adaptation. Like *D. ochrophaeus* [17] and *P. signifer* [14], the degree of female discrimination against foreign males in *T. septentrionalis* depends on how geographically and genetically separated populations are from one another (Table 1; Figs. 3a, b). This finding raises the question of how females distinguish between native males and those from other populations separated by various distances to their own. In order to answer this question, we discuss below the implications of our results for the roles of individual factors in mate assessment by female northern grass lizards and their relative contributions to population divergence in female mate preferences.

Our data showed a strong concordance between the extent of geographical isolation among the five populations and the degree of genetic differentiation (Fig. 4), supporting a widely accepted idea that more distant allopatry should reflect reduced rates of contemporary gene flow and thus increased genetic differentiation [9, 14, 17]. More distantly allopatric populations of *T. septentrionalis* have undergone greater divergence in female mate preferences associated with longer periods of genetic isolation [27]. This was evidenced by the ability of LS females to discriminate more strongly against males from populations geographically and genetically more distant to their own (Fig. 2b). However, our SEM analysis shows that genetic differentiation is not the direct determinant of population divergence in female mate preferences in *T. septentrionalis*. Instead, genetic differentiation influences female mate preference largely through its influence on morphological divergence (Fig. 4).

Like other lizards of diverse taxa including gekkonid [37], lacertid [38, 39], phrynosomatid [40] and scincid [41] species, morphological differences in *T. septentrionalis* are coupled with environmental differentiation [42], which is strongly concordant with geographical distance between populations (Fig. 4). Our morphometric analysis shows that male *T. septentrionalis* from genetically and geographically more proximate populations are more similar in appearance (Fig. 2d). Our SEM analysis further shows that male morphological divergence among populations reflects the combined effect of environmental and genetic differences and is of primary importance for population divergence in female mate preferences (Fig. 4). It worth noting, however, that a male’s individual morphological traits such as body size and head size do not seem to influence mate assessment by female *T. septentrionalis*, as revealed by the following lines of evidence.

Like most lizards, the body posture (male grasps one side of female’s body with teeth, and then male-female cloacal regions come into contact) of mating partners [including heterospecific [(between *T. septentrionalis* (male) and *T. sexlineatus* (female); Fig. 1a] and conspecific homosexual (between two males of *T. septentrionalis*; Fig. 1b) mating partners] in *T. septentrionalis* was primarily determined by the male’s posture, remained nearly unchanged throughout the mating process and could not be achieved without relying on the male’s bite force (Fig. 1). Bite forces are greater in lizards with larger heads [43, 44], and it is generalizable to lizards including *T. septentrionalis* that head size positively correlates with body size [45]. Thus, if any mating-related behavior (male sexual coercion, female receptivity, female proceptivity, or mate quality assessment by females) were associated with the body size, head size and thus bite force of males, mating latency and/or copulation duration would have been expected to differ among females mating with males from different populations (Additional file 2). Nevertheless, such differences were not observed in this study (Table 1), thus allowing us to conclude that female *T. septentrionalis* use the male’s overall body appearance rather than individual morphological traits as cues or signals to recognize and mate assortatively according to their own habitat or population. This conclusion is consistent with that drawn for *P. signifer* where females prefer to mate with males from the native or nearby population also because of morphological and genetic similarities between closely related populations [14, 46]. Mating with morphologically similar or familiar males could be a way of reinforcing local adaptation to diverse environmental conditions, avoiding homogenizing differences between allopatric populations and optimizing the local genotype of the offspring [15, 20, 47]. This is especially true for philopatric species such as *T. septentrionalis* [48], or for species such as most widespread *Takydrumus* lizards that occupy heterogeneous ranges, where offspring are likely to breed in the same habitat as their parents [20, 38, 49, 50].

## Conclusions

In this study, we offered female *T. septentrionalis* from one population a choice of males from their own population and another four populations to test whether the preferences that females display in the mate choice trials correlate with phenotypic adaptation to local environments, or to the neutral genetic distance measured by divergence of mitochondrial DNA sequence loci. Our results show that female preference for males from their own population can be interpreted as population dependent assortative mating and generally support the hypothesis that local adaptation rather than neutral genetic distance influences female mate preference in *T. septentrionalis*. Female northern grass lizards judge native versus foreign mates by appearance. Our results highlight the importance of comprehensive studies integrating ecological, molecular and experimental behavior approaches to understand population divergence in mate preferences as a consequence of local adaptations, which can be a first and crucial step during the process of speciation. Future work could usefully investigate other visual signals and chemical signals to determine whether factors other than body shape may also contribute the decision process of *T. septentrionalis*. It has been found that *Takydromus* lizards among species having the visual [51] and chemical [52–55] communication systems and abilities to signal with conspecifics and alter their behaviour.

## Supplementary information

**Additional file 1: Figure S1.** Photos showing the three distinct color morphs. Morph 1 is shared by both females (a1) and males (a2), morph 2 is a typical male coloration (b), and morph 3 is a typical female coloration (c). Red, white and black colors on the head are not natural but temporary marks

**Additional file 2: Figure S2.** Descriptive statistics, expressed as mean (for SVL) or SVL-specific mean (for AL, HL and HW) values + SE, for adult males collected from the Lishui (LS), Liuheng (LH), Xiushan (XS), Chuzhou (CZ) and Chang’an (CA) populations. Mean or SVL-specific mean values with different letters differed significantly (Tukey’s post hoc test, α = 0.05). All units are in mm.

**Additional file 3: Table S1.** Loading of the first two axes of a principal component analysis on 22 geographical and climatic variables. Variables with the main contribution to each factor are in bold face font.

**Additional file 4: Table S2.** Loading of the first two axes of a principal component analysis on three size-adjusted morphological parameters, on which size effects were removed by using residuals from the regressions on snout-vent length. Variables with the main contribution to each factor are in bold face font.

**Additional file 5: Table S3.** Results of the structural equation modeling for the relative contributions of four factors to female mate preference.

## Data Availability

All data generated or analyzed during this study are included in this published article and its supplementary information files (Additional files 1, 2, 3, 4 and 5).

## Abbreviations

AL: Abdomen (or axillo-groin) lengt
ANOVA: Analysis of variance
ANCOVA: Analysis of covariance
CZ: Chuzhou
CA: Chang’an
HL: Head length
HW: Head width
LH: Liuheng
LS: Lishui
PCA: Principal components analysis
SEM: Structural equation modelling
SVL: Snout-vent length
XS: Xiushan
