# Supplementary figures and images for: Female northern grass lizards judge mates by body shape to reinforce local adaptation
Source: Front Zool. 2020 Aug 4;17:22. doi: 10.1186/s12983-020-00367-9 (PMC7409496; doi:10.1186/s12983-020-00367-9)

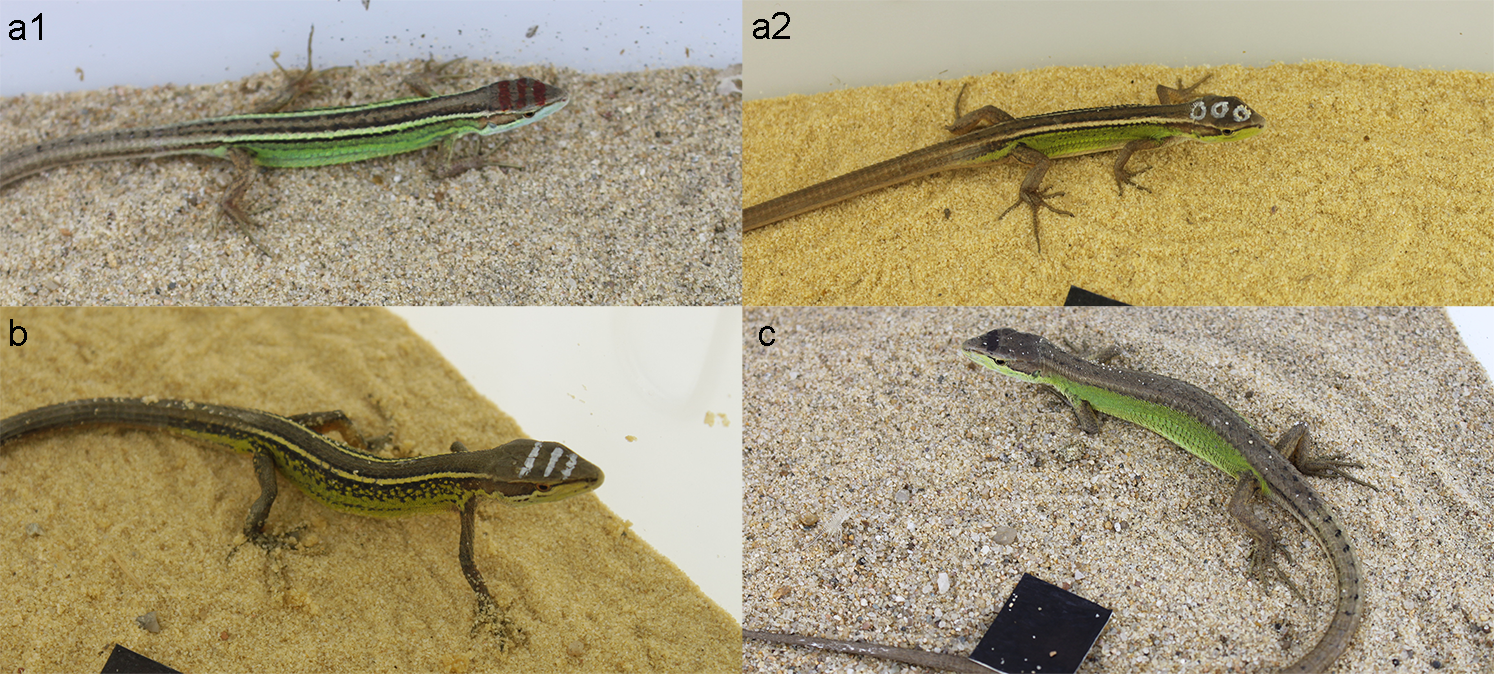

Supplement: Supplementary file 1 — Additional file 1: Figure S1. Photos showing the three distinct color morphs. Morph 1 is shared by both females (a1) and males (a2), morph 2 is a typical male coloration (b), and morph 3 is a typical female coloration (c). Red, white and black colors on the head are not natural but temporary marks [file 12983_2020_367_MOESM1_ESM.tif]

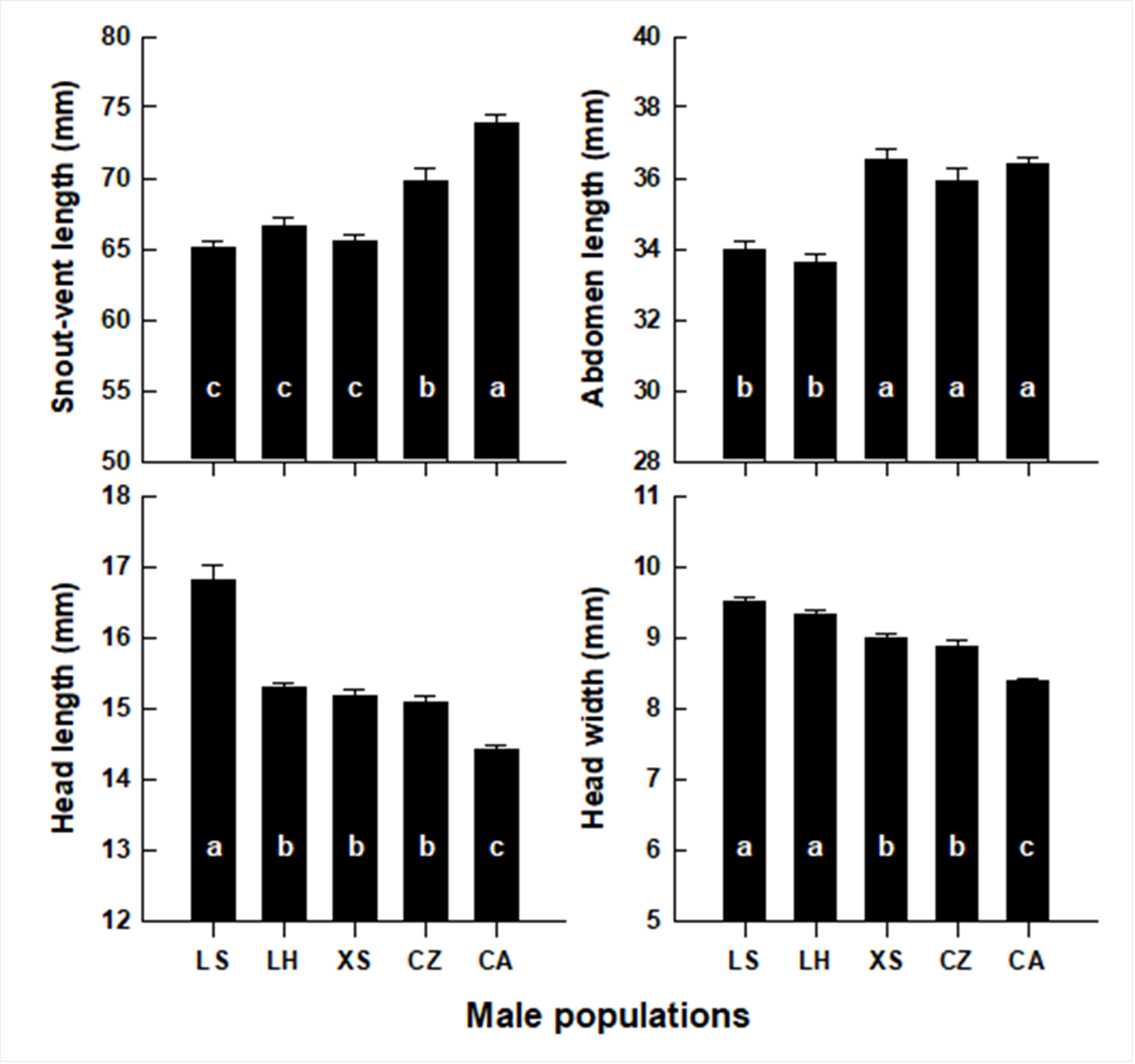

Supplement: Supplementary file 2 — Additional file 2: Figure S2. Descriptive statistics, expressed as mean (for SVL) or SVL-specific mean (for AL, HL and HW) values + SE, for adult males collected from the Lishui (LS), Liuheng (LH), Xiushan (XS), Chuzhou (CZ) and Chang’an (CA) populations. Mean or SVL-specific mean values with different letters differed significantly (Tukey’s post hoc test, α = 0.05). All units are in mm. [file 12983_2020_367_MOESM2_ESM.tif]
