# Supplementary material for: Female northern grass lizards judge mates by body shape to reinforce local adaptation
Source: Front Zool. 2020 Aug 4;17:22. doi: 10.1186/s12983-020-00367-9 (PMC7409496; doi:10.1186/s12983-020-00367-9)
Supplement: Supplementary file 3 — Additional file 3: Table S1. Loading of the first two axes of a principal component analysis on 22 geographical and climatic variables. Variables with the main contribution to each factor are in bold face font. [file 12983_2020_367_MOESM3_ESM.doc]

**Table S1** Loading of the first two axes of a principal component analysis on 22 geographical and climatic variables. Variables with the main contribution to each factor are in bold face font.

|  | PC1 | PC2 |
| --- | --- | --- |
| Latitude | **0.938** | 0.104 |
| Longitude | **−0.980** | 0.032 |
| Altitude | **0.88** | 0.182 |
| Annual mean temperature (Bio1) | **−0.968** | −0.181 |
| Mean diurnal range (Bio2) | **0.727** | −0.681 |
| Isothermality (Bio3) | 0.583 | **−0.730** |
| Temperature seasonality (Bio4) | **0.711** | −0.538 |
| Max temperature of warmest month (Bio5) | **−0.809** | −0.532 |
| Min temperature of coldest month (Bio6) | **−0.994** | 0.104 |
| Temperature annual range (Bio7) | **0.779** | −0.611 |
| Mean temperature of wettest quarter (Bio8) | −0.337 | −0.642 |
| Mean temperature of driest quarter (Bio9) | **−0.990** | 0.129 |
| Mean temperature of warmest quarter (Bio10) | **−0.873** | −0.344 |
| Mean temperature of coldest quarter (Bio11) | **−0.999** | 0.014 |
| Annual precipitation (Bio12) | **−0.901** | −0.203 |
| Precipitation of wettest month (Bio13) | −0.502 | **−0.862** |
| Precipitation of driest month (Bio14) | **−0.981** | 0.189 |
| Precipitation seasonality (Bio15) | **0.903** | −0.428 |
| Precipitation of wettest quarter (Bio16) | −0.473 | −0.789 |
| Precipitation of driest quarter (Bio17) | **−0.993** | 0.119 |
| Precipitation of warmest quarter (Bio18) | −0.594 | −0.762 |
| Precipitation of coldest quarter (Bio19) | **−0.994** | 0.077 |
| Variance explained (%) | 70.1 | 21.8 |
