# Supplementary material for: Female northern grass lizards judge mates by body shape to reinforce local adaptation
Source: Front Zool. 2020 Aug 4;17:22. doi: 10.1186/s12983-020-00367-9 (PMC7409496; doi:10.1186/s12983-020-00367-9)
Supplement: Supplementary file 4 — Additional file 4: Table S2. Loading of the first two axes of a principal component analysis on three size-adjusted morphological parameters, on which size effects were removed by using residuals from the regressions on snout-vent length. Variables with the main contribution to each factor are in bold face font. [file 12983_2020_367_MOESM4_ESM.doc]

**Table S2** Loading of the first two axes of a principal component analysis on three size-adjusted morphological parameters, on which size effects were removed by using residuals from the regressions on snout-vent length. Variables with the main contribution to each factor are in bold face font.

|  | PC1 | PC2 |
| --- | --- | --- |
| Abdomen length | **−0.733** | **−**0.643 |
| Head length | **0.764** | **−**0.594 |
| Head width | **0.923** | **−**0.019 |
| Variance explained (%) | 65.8 | 25.6 |
