# Supplementary material for: Female northern grass lizards judge mates by body shape to reinforce local adaptation
Source: Front Zool. 2020 Aug 4;17:22. doi: 10.1186/s12983-020-00367-9 (PMC7409496; doi:10.1186/s12983-020-00367-9)
Supplement: Supplementary file 5 — Additional file 5: Table S3. Results of the structural equation modeling for the relative contributions of four factors to female mate preference. [file 12983_2020_367_MOESM5_ESM.doc]

**Table S3** Results of the structural equation modeling for the relative contributions of four factors to female mate preference.

|  | Geographical distance | Genetic differentiation | Environmental dissimilarity | Morphological difference |
| --- | --- | --- | --- | --- |
| Standardized direct effects | | | | |
| Genetic differentiation | 0.69 | 0.00 | 0.00 | 0.00 |
| Environmental dissimilarity | 0.55 | 0.00 | 0.00 | 0.00 |
| Morphological difference | 0.00 | −0.93 | −0.03 | 0.00 |
| Female preference | 0.00 | 0.00 | 0.00 | 0.97 |
| Standardized indirect effects | | | | |
| Genetic differentiation | 0.00 | 0.00 | 0.00 | 0.00 |
| Environmental dissimilarity | 0.00 | 0.00 | 0.00 | 0.00 |
| Morphological difference | −0.66 | 0.00 | 0.00 | 0.00 |
| Female preference | −0.63 | −0.90 | −0.30 | 0.00 |
| Standardized total effects | | | | |
| Genetic differentiation | 0.69 | 0.00 | 0.00 | 0.00 |
| Environmental dissimilarity | 0.55 | 0.00 | 0.00 | 0.00 |
| Morphological difference | −0.66 | −0.93 | −0.03 | 0.00 |
| Female preference | −0.63 | −0.90 | −0.30 | 0.97 |
